# Focused molecular analysis of small cell lung cancer: feasibility in routine clinical practice

**DOI:** 10.1186/s13104-015-1675-x

**Published:** 2015-11-18

**Authors:** Fatma Abdelraouf, Adam Sharp, Manisha Maurya, Debbie Mair, Andrew Wotherspoon, Alex Leary, David Gonzalez de Castro, Jaishree Bhosle, Ayatallah Nassef, Taghrid Gaafar, Sanjay Popat, Timothy A. Yap, Mary O’Brien

**Affiliations:** Lung Cancer Unit, Department of Medicine, Royal Marsden NHS Foundation Trust, Downs Road, Sutton, London, SM2 5PT UK; Clinical Pathology Department, Faculty of Medicine, Cairo University, Cairo, Egypt; The Institute of Cancer Research, London, UK; National Heart and Lung institute, London, UK

**Keywords:** Small cell lung cancer, Genomic aberration, Molecular profiling, *EGFR*, *KRAS*, *BRAF*, *NRAS*, *ALK*, *MET*

## Abstract

**Background:**

There is an urgent need to identify molecular signatures in small cell lung cancer (SCLC) that may select patients who are likely to respond to molecularly targeted therapies. In this study, we investigate the feasibility of undertaking focused molecular analyses on routine diagnostic biopsies in patients with SCLC.

**Methods:**

A series of histopathologically confirmed formalin-fixed, paraffin-embedded SCLC specimens were analysed for epidermal growth factor receptors (*EGFR*)*, KRAS, NRAS and BRAF* mutations, *ALK* gene rearrangements and *MET* amplification. *EGFR* and *KRAS* mutation testing was evaluated using real time polymerase chain reaction (RT-PCR cobas^®^), *BRAF* and *NRAS* mutations using multiplex PCR and capillary electrophoresis-single strand conformation analysis, and *ALK* and *MET* aberrations with fluorescent in situ hybridization. All genetic aberrations detected were validated independently.

**Results:**

A total of 105 patients diagnosed with SCLC between July 1990 and September 2006 were included. 60 (57 %) patients had suitable tumour tissue for molecular testing. 25 patients were successfully evaluated for all six pre-defined molecular aberrations. Eleven patients failed all molecular analysis. No mutations in *EGFR*, *KRAS* and *NRAS* were detected, and no *ALK* gene rearrangements or *MET* gene amplifications were identified. A V600E substitution in *BRAF* was detected in a Caucasian male smoker diagnosed with SCLC with squamoid and glandular features.

**Conclusion:**

The paucity of patients with sufficient tumour tissue, quality of DNA extracted and low frequency of aberrations detected indicate that alternative molecular characterisation approaches are necessary, such as the use of circulating plasma DNA in patients with SCLC.

## Background

Lung cancer is the leading cause of cancer-related deaths globally [1]. Small cell lung cancer (SCLC) is an aggressive neuroendocrine subtype of lung cancer that represents 13–15 % of all lung cancers [2]. SCLC is strongly linked to smoking and is characterised by rapid doubling time with early onset of dissemination and frequently presents (60–70 %) with advanced disease extensive stage (ED) [3]. Extensive stage disease SCLC has a median overall survival of 7–12 months [4]. The mainstay of treatment remains platinum-based doublet chemotherapy and despite benefits from prophylactic cranial radiation (PCI) and thoracic radiotherapy systemic treatments have not changed over the last few decades [5–7]. SCLC demonstrates impressive response rates to first-line chemotherapy but relapse with chemotherapy resistant disease and death frequently shortly follows [8]. There thus remains an urgent clinical need to develop novel therapeutic strategies that impact on this common, aggressive disease.

Numerous unselected phase II clinical trials have evaluated molecularly targeted therapies in first-line, maintenance and second-line treatment of SCLC [3, 9–12]. These have included agents that regulate a plethora of cellular processes including angiogenesis, cell signaling, DNA repair pathways and apoptosis. No targeted therapy has demonstrated efficacy is SCLC and no targeted treatments have been licenced for use in this aggressive disease. Molecular aberrations have been identified in patients with non-small cell lung cancer (NSCLC) over the past 10 years and revolutionised treatment strategies [13]. Until recently little was known about the molecular profile of SCLC. Recent studies have begun to unravel molecular aberrations present in patients with SCLC [2, 14–18]. Two studies used targeted gene panels and identified genetic aberrations in 15 % (9 gene panel) and 6.2 % (6 gene panel) of patients with SCLC [2, 15]. The most common aberrations within the targeted studies were phosphatidylinositol-3-kinase catalytic subunit alpha (*PI3KCA* 15 %) and *MET* (4.4 %) [2, 15]. A more comprehensive analysis of 236 cancer genes using next generation sequencing demonstrated all 98 patients to have at least one genomic alteration [14]. The most common aberrations were rapamycin-insensitive companion of mTOR (*RICTOR*; 10 %), stem cell factor receptor tyrosine kinase (*KIT*; 7 %), *PI3KCA* (6 %) epidermal growth factor receptor (*EGFR*; 5 %), phosphatase and tensin homolog (*PTEN*; 5 %) and *KRAS* (5 %) [14]. The identification and validation of molecular aberrations within SCLC may potentially facilitate the development of effective targeted therapies for the treatment of SCLC.

We investigated the feasibility of performing molecular studies on biopsy material surplus to the SCLC diagnostic algorithm. We assessed the mutational status of several oncogenes (*EGFR*, *BRAF*, *KRAS* and *NRAS*), together with *ALK* gene rearrangements and *MET* gene amplification in patients with SCLC.

## Methods

### Patient cohort

**Fig. 1:**
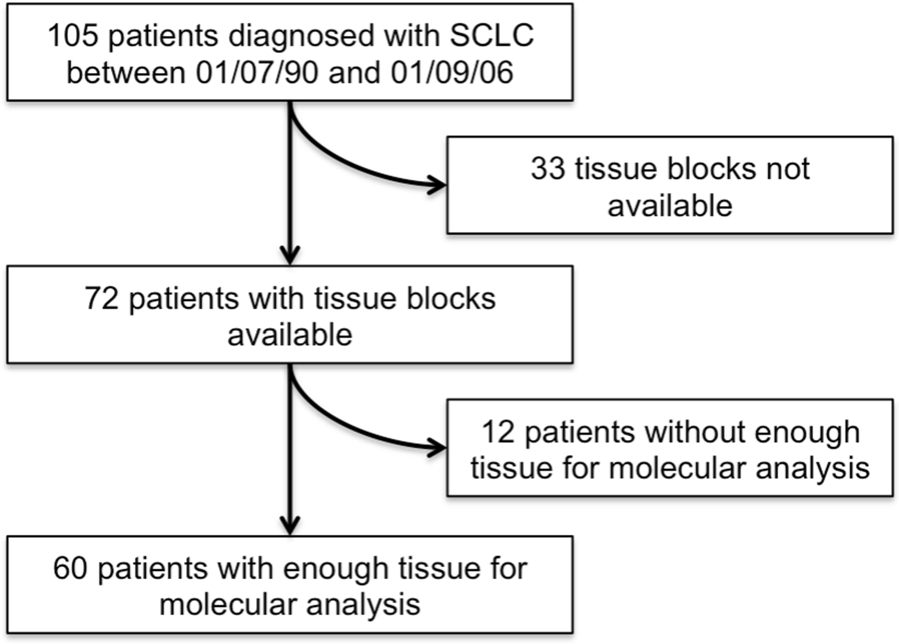
Patient cohort. One hundred and five patients were diagnosed with SCLC between the 1st July 1990 and 1st September 2006. Thirty-three patients identified had no tissue blocks available and a further 12 patients had insufficient tissue for molecular analysis. Sixty patients with sufficient tissue for molecular analysis were included in the study

One hundred and five patients were diagnosed with SCLC between 1st July 1990 and 1st September 2006 at the Royal Marsden Hospital. Seventy-two patients had formalin-fixed, paraffin-embedded (FFPE) blocks of which 60 were deemed to have enough tissue for molecular analysis (Fig. 1). Patients included within the study had a biopsy-proven diagnosis of SCLC and were ≥18 years old. Patients were excluded if they only had a cytological or clinical diagnosis of SCLC. Informed consent for use of tissue for research was obtained if the patient was still alive and diagnosed after September 1st 2006, according to the Human Tissue Act. The study was approved by both the Research Ethics Committee (11/SC/0073) and Local Committee for Clinical Research (CCR 3428). All samples were routinely fixed. The hospitals electronic patient records were used to collect clinical characteristics including age, sex, smoking history, performance status (PS), stage (VALG), type of treatment and first-line chemotherapy regime received (Table 1). Smoking status for patients was defined at diagnosis. These were divided into current smokers and ex-smokers depending on their smoking status at diagnosis.

**Table 1:** Patient demographics

	N = 60	%
Mean age	65.9	
Range	45–84	
Gender		
Male	29	48.3
Female	31	51.7
Smoking history		
Current smoker	44	73.3
Past smoker	16	26.7
ECOG PS		
0	5	8.3
1	15	25
2	20	33.3
3	19	31.7
4	1	1.7
Stage		
LD	33	55
ED	27	45
Histology		
Small cell carcinoma	59	98.3
Combined small cell carcinoma	1	1.7
Treatment		
Chemotherapy	51	85
Refused	3	5
Not fit	3	5
RT/surgery	3	5
First-line chemotherapy		
Carbo/etoposide	13	21.7
ACE	13	21.7
MVP	13	21.7
Etoposide	5	8.3
Cisplatin/etoposide	2	3.3
Other/no chemotherapy	5/9	8.3/15
Number of cycles of treatment		
1–3 cycles	16	31.4
4–6 cycles	35	68.6
Response to first-line treatment		
Complete response	4	7.8
Partial response	29	56.9
Stable disease	10	19.6
Progressive disease	8	15.7

*ECOG* eastern cooperative oncology group, *PS* performance status, *LD* limited disease, *ED* extensive disease, *ACE* adriamycin, cyclophosphamide and etoposide, *MVP* mitomycin C, vinblastine and cisplatin

SCLC diagnosis was not validated for the purposes of this study. It had previously been made by pathologists at the Royal Marsden Hospital according to the 2004 World Health Organisation classification based on morphology (uniform round to spindle-shaped small cells, sparse cytoplasm, high mitotic index and necrotic areas). Presence of cancer cells within biopsies was confirmed by a histopathologist (AW) prior to molecular analysis.

### Molecular characterisation

#### *DNA* preparation

For all suitable tumour specimens, 5 and 2 μm tissue section slides were prepared. The 5 μm slides were used for DNA extraction and subsequent analysis for *EGFR*, *KRAS*, *BRAF* and *NRAS* while the 2 μm slides were used directly for fluorescent in situ hybridisation (FISH) analysis of *ALK* and *MET*. DNA extraction was done using Roche cobas^®^ DNA sample preparation kit (Roche Molecular Systems Incorporation, Branchburg, New Jersey, USA).

#### *EGFR* mutation

EGFR mutation analysis was performed on extracted DNA using Roche cobas^®^*EGFR* Mutation test (Roche Molecular Systems Incorporation, Branchburg, New Jersey, USA). It is a CE-marked allele-specific real-time PCR assay designed to detect mutations in exons 18, 19, 20, and 21 of the *EGFR* gene in FFPE tissue specimens. The *EGFR* test is a 3-tube assay designed to detect G719X (G719A, G719C, and G719S), in exon 18, deletions and complex mutations in exon 19, S768I, T790M, and insertions in exon 20, and L858R in exon 21. Mutation detection is achieved through PCR analysis with the cobas^®^ 4800 System (Roche Molecular Systems Incorporation, Branchburg, New Jersey, USA) with automated result interpretation software. The results were presented in the form of positive, negative, invalid or failed.

#### *KRAS* mutation

*KRAS* mutation analysis was performed on extracted DNA by the cobas^®^*KRAS* Mutation test (Roche Molecular Systems Incorporation, Branchburg, New Jersey, USA). It is a CE-marked real-time TaqMelt PCR assay designed to detect somatic mutations in codons 12, 13 (exon 2) and 61 (exon 3) of the *KRAS* gene. Mutations are detected by melting curve analysis, using the cobas^®^ 4800 System (Roche Molecular Systems Incorporation, Branchburg, New Jersey, USA) with automated result interpretation software. The results were presented in the form of positive, negative, invalid or failed.

#### *BRAF* and *NRAS* mutation

*BRAF* and *NRAS* mutation analysis was performed on extracted DNA by multiplex PCR and capillary electrophoresis-single strand conformation analysis (CE-SSCA) using an in-house procedure. PCR was done using the 2720 Thermal Cycler (Applied Biosystems^®^, Foster City, USA) and the *BRAF/NRAS* mutational analysis was done using the ABI 3130XL Genetic Analyzer (Applied Biosystems^®^, Foster City, USA). For *BRAF*, a V600E mutation will result in a characteristic pattern in the CE-SSCA at all different temperatures and therefore requires no further confirmation by sequencing. For any unclear results, Direct DNA Sanger sequencing was performed for confirmation. For *NRAS*, any mobility shift identified by CE-SSCA in codon 61 or codon 12/13 was confirmed by Direct DNA Sanger sequencing. This was done using the 9800 fast thermal cycler (Applied Biosystems^®^, Foster City, USA) and the ABI 3500 Genetic analyzer (Applied Biosystems^®^, Foster City, USA). Sequencing data was analysed using Mutation Surveyor software.

#### *ALK* rearrangements

FISH analysis was directly performed on the 2 μm slides. Vysis LSI *ALK* Dual Colour Break Apart FISH Probe (Abbott Molecular Inc., Des Plaines, IL, USA) was used for *ALK* testing (Abbott Vysis *ALK* Break Apart FISH Probe Kit, Abbott laboratories, Illinois, USA). Results were analysed using Zeiss AxioImager Z2 Fluorescence microscope (Carl Zeiss Microscopy, Madrid, Spain). Reporting of *ALK* FISH test results was done according to the FISH probe manufacturers (Abbott laboratories, Illinois, USA) with the normal cut-off level of 15 % in FFPE tissue specimen.

#### *MET* amplification

For *MET* amplification detection by FISH, KREATECH Poseidon™ Repeat Free™ C-*MET* (7q31) and SE7 probe (KREATECH diagnostics, Amsterdam, Holland) was used. Results were analysed using Zeiss AxioImager Z2 Fluorescence microscope (Carl Zeiss Microscopy, Madrid, Spain). Reporting of *MET* FISH test results has no standardized procedure as *MET* FISH is not used as a diagnostic tool. In our study, the reporting was done by using 10 % as a cut-off similar to *ALK* amplification [19]. If 10 % or more of the cells show a ratio of R: G >2, then the sample is considered *MET* positive. All samples with more than 5 % *MET* positivity were re-scored using the PathVysion scoring system, similar to the approved *HER2* amplification scoring system in breast cancer. The PathVysion scoring system was done by determining the ratio between the red signals and the green signals (adding the total number of reds in the 20 cells (R) and the total number of greens in 20 cells (G), then dividing R/G and getting a ratio). If the ratio is <2, the sample does not show *MET* amplification and if the ratio is ≥2, then the sample shows *MET* amplification (as per PathVysion HER-2 DNA Probe Kit, Abbott laboratories, Illinois, USA).

## Results

### Patient characteristics

Between 1st July 1990 and 1st September 2006 FFPE blocks for 60 patients were assessed for genomic aberrations. The patient characteristics are shown in Table 1. The mean age (range) was 65.9 (45–84) years. Twenty-nine patients (48.3 %) diagnosed with SCLC were male and 31 patients (51.7 %) were female. Forty-four patients (73.3 %) were current smokers and 16 patients (26.7 %) were past smokers. There were no never smokers identified within this study. Forty patients (66.7 %) were Eastern Cooperative Oncology Group (ECOG) PS 0–2. A total of 59 patients (98.3 %) were diagnosed with SCLC and one patient (1.7 %) was diagnosed with combined SCLC. Those biopsies in which immunohistochemistry had been performed historically were recorded from our electronic patient records (Table 2). Twenty-seven patients (45 %) had ED SCLC at diagnosis and 51 patients (85 %) received first-line chemotherapy. Thirty-five patients (68.6 %) received 4–6 cycles of chemotherapy whist 16 patients (31.4 %) received 1–3 cycles. The response rates to first-line chemotherapy were complete response (4 patients 7.8 %), partial response (29 patients 56.9 %), stable disease (10 patients 19.6 %) and progressive disease (8 patients 15.7 %).

**Table 2:** Results of immunohistochemistry analysis (historical)

Immunohistochemical test	N = 60
TTF1	
Positive	5
Negative	0
Not available	55
CD56	
Positive	4
Negative	1
Not available	55
CAM5.2	
Positive	33
Negative	3
Not available	24
Cytokeratin 7	
Positive	2
Negative	0
Not available	58
Chromogranin A	
Positive	15
Negative	13
Not available	32
NSE	
Positive	14
Negative	5
Not available	41
Synaptophysin	
Positive	21
Negative	12
Not available	27
CD45	
Positive	0
Negative	31
Not available	29
MNF116	
Positive	27
Negative	4
Not available	29
EMA	
Positive	18
Negative	8
Not available	34

### Molecular characterisation

#### Sample quality

Entry into the study was determined by the presence of cancer cells (morphological analysis) and sufficient FFPE tissue for molecular analysis (determined by AW). Samples were not pre-assessed for their quality and yield of DNA prior to study analysis. Therefore a number of samples had insufficient DNA (quantity and/or quality) for molecular analysis (successful analysis summarised in Table 3). Twenty-five cases (41.7 %) were successfully analysed for all mutations (*EGFR*, *KRAS*, *BRAF* and *NRAS*), gene rearrangement (*ALK*) and amplification (*MET*) (Table 3). There was marked variation in the DNA concentration of these samples (0.2–57.3 ng/µl; mean 12.1 ng/µl). Thirty cases (50 %) were successfully analysed for all mutations (*EGFR*, *KRAS*, *BRAF* and *NRAS*) (Table 3). These samples also showed marked variation in extracted DNA concentration (1.0–57.3 ng/µl; mean 11.0 ng/µl). Finally, 11 cases (18.3 %) provided no interpretable results for all mutations with DNA concentrations ranging from 0.20 to 31.7 ng/µl; mean 9.8 ng/µl) (Table 3). FISH analysis was successful for *ALK* rearrangement and *MET* amplification in 58 (96.7 %) and 42 (70 %) of SCLC cases (Table 3).

**Table 3:** Results of successful molecular analysis

Gene	
BRAF	
Aberration	1 (*BRAF V600E*)
No aberration	46
EGFR	
Aberration	0
No aberration	31
KRAS	
Aberration	0
No aberration	35
NRAS	
Aberration	0
No aberration	37
ALK	
Rearrangement	0
No rearrangement	58
MET	
Amplification	0
No amplification	42

#### Genomic aberrations

**Fig. 2:**
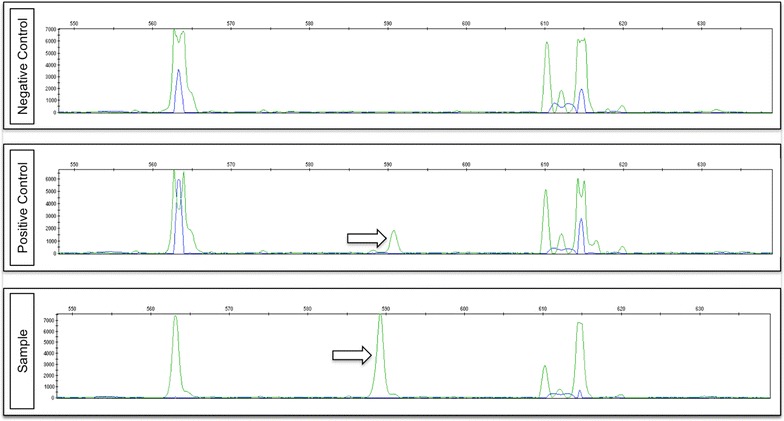
Positive BRAF mutation detected by capillary electrophoresis-single strand conformation analysis. Capillary electrophoresis-single strand conformation analysis demonstrates V600E BRAF mutation in patient sample. Positive and negative controls are shown. *Black arrow* corresponds to extra peak representing the V600E BRAF mutation

We detected a single genetic aberration within the 60 cases of SCLC analysed. This was within the BRAF gene. The mutation was a single amino acid substitution of valine (V) to glutamic acid (E) at position 600 (V600E mutation) (Fig. 2; Table 3). In cases that were suitable for analysis there were no mutations identified within the *EGFR* (31 cases), *KRAS* (35 cases) and *NRAS* (37 cases) genes (Table 3). In addition, there were no cases identified with *ALK* rearrangements (58 cases) or *MET* amplification (42 cases) (Table 3). The patient identified with a BRAF V600E substitution was a 55 year-old Caucasian current smoker. He was diagnosed, radiologically, with a T2N0M0 lung cancer and underwent lobectomy. His postoperative histology demonstrated combined small cell carcinoma with squamoid and glandular features. He received no adjuvant therapy. Six months later he presented with recurrent LD SCLC for which he received radical chemoradiotherapy (four cycles of Carboplatin/Etoposide and radiotherapy 36 Gy in 12 fractions) and PCI with good response. He died suddenly 9 months later.

#### Immunohistochemical analysis

Repeat immunohistiochemistry (IHC) was not performed on patient samples in this study. Results for the 42 patients who had historical IHC performed were collated from our electronic patient records system (Table 2). Five out of five patients (100 %) stained positive for TTF-1 and 15 out 28 patients (53.6 %) stained positive for Chromagranin A (Table 2). CD56 and synaptophsin were expressed in 4 out of 5 (80 %) and 21 out of 33 (63.6 %) respectively (Table 2).

## Discussion

In the present study, the mutation rate of a 4-gene panel (*EGFR*, *NRAS*, *KRAS* and *BRAF*), *ALK* gene rearrangements and *MET* amplification was evaluated in patients with SCLC who had enough tissue for molecular analysis after diagnosis between the 1st July 1990 and 1st September 2006 (Fig. 1). This study explores the feasibility of performing focused molecular analysis within standard diagnostic algorithms in SCLC.

Sixty (57 %) patients of the 105 diagnosed with SCLC in the study period had sufficient tissue for genomic analysis and 45 patients (33 patients no tissue block; 12 patients insufficient tissue) lacked sufficient tissue for molecular analysis (Fig. 1). Twenty-five (42 %) patients were successfully analysed for all molecular aberrations tested (*EGFR*, *NRAS*, *KRAS* and *BRAF* mutations; *ALK* rearrangements; *MET* amplification). A single mutation (1.7 % of patients) within *BRAF* was identified. The mutation was a single amino acid substitution of valine (V) to glutamic acid (E) at position 600 (V600E mutation). This was detected in a Caucasian male smoker diagnosed with SCLC with squamoid and glandular features. It is conceivable that the mutation was within the squamoid component although this would still represent a rare event within NSCLC [20].

The failure rates observed may be, in part, due to the paucity of tissue available from routine diagnostic biopsies. Routinely the diagnosis of SCLC is made without immunohistochemistry analysis. However in this study 42 patients had further immunostaining performed due to diagnostic uncertainty (Table 2). Consistent with other studies these confirmed high positivity for TTF-1, cytokeratin, CAM, CD56, NSE, chromogranin, synoptophysin and EMA [21, 22]. These extra analyses compound further the paucity of patient tissue available for molecular analysis.

The quality of extracted DNA from tissue biopsies may account for the failure rates observed. Thirty (50 %) of the 60 patients evaluated were successfully analysed for *EGFR*, *NRAS*, *KRAS* and *BRAF* mutations. The concentration of extracted DNA in these cases was 1.0–57.3 ng/µl (mean 11.0 ng/µl). Interestingly, in the eleven (18.3 %) patients that failed molecular analysis for *EGFR*, *KRAS*, *BRAF* and *NRAS* mutations the extracted DNA concentrations (0.2–31.7 ng/µl; mean 9.8 ng/µl) did not differ from those patients successfully analysed for molecular aberrations. This suggests that DNA quality as opposed to DNA quantity is critically important for success of the molecular analysis performed within this study.

Two recent studies used focused gene panels to identify molecular aberrations in SCLC patients [2, 15]. The most common alterations identified were in PIK3CA (5 % mutations and 10 % amplifications) and MET (4.4 % mutation) [2, 15]. Similar to our own study the incidence of *EGFR* (1.8 % and 1.7 %), *NRAS* (0 %) and *KRAS* (0 % and 1.7 %) mutations, *ALK* rearrangements (0 %) and MET amplifications (1.7 %) were low or not seen [2, 15]. In contrast to our own there were no cases (173 patients) of SCLC with BRAF mutations identified in these studies. These studies along with our own study have attempted to identify potential molecular alterations that could stratify patients to targeted therapies. However, the alterations identified were of low frequency (1.8, 6.1 and 15 % of patients analysed) and alternative approaches should be sought.

Recent advances in cancer genome sequencing technologies have led to more comprehensive analysis of SCLC through whole-exome sequencing and targeted sequencing [14, 16–18]. These studies have shown high prevalence of inactivating mutations within *TP53* and *Rb* tumour suppressor genes, amplification of *MYC* family members and mutation of histone modifiers (*CREBBP* and *EP300*) [14, 16–18]. Ross and colleagues identified 53 % of SCLC cases with at least a single actionable alteration with the potential to personalise therapy [14]. These next generation sequencing approaches are likely to be more fruitful than focused approaches in driving translational studies within SCLC.

**Fig. 3:**
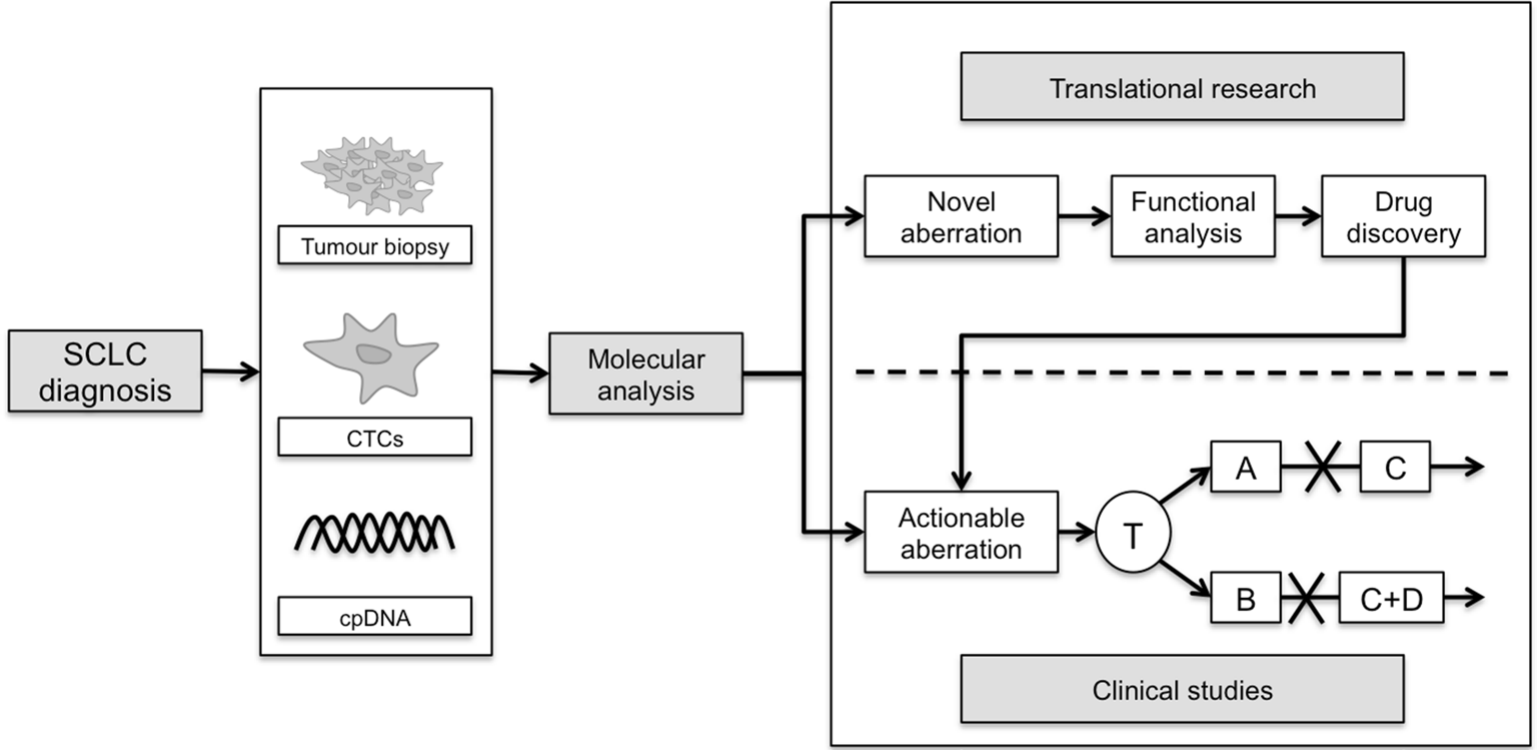
Proposed model for molecular analysis of SCLC and incorporation into translational studies. Patients should undergo a tumour biopsy to obtain sufficient tissue for molecular analysis alongside collection of blood specimens to isolate circulating plasma DNA (cpDNA) and circulating tumour cells (CTCs). Molecular analysis of these samples should be used for translational studies. These may be clinical studies in which patients identified to have actionable aberrations enter clinical studies in which there treatment (T) is determined by sequencing data. These patients should have sequential genomic analysis through cpDNA and CTCs to identify further molecular aberrations (X) that may confer resistance and determine further treatment (A, B, C and D) strategies. These molecular studies are also likely to identify novel or non-actionable changes within SCLC that should be further studied to determine their functional role and potential as novel therapeutic targets for the treatment of SCLC

Our study demonstrates the limitations of using traditional diagnostic biopsies for molecular analysis in SCLC. In addition to difficulties with tissue availability, this and other focused studies using pre-defined gene panels have identified low frequency of actionable mutations in patients with SCLC. Moreover, small tumour biopsies contain relatively few tumour cells limiting our understanding of the heterogeneity of SCLC. We propose a model for future translational studies in SCLC whereby patients undergo tumour biopsies at diagnosis to obtain sufficient tissue for molecular analysis (Fig. 3). Blood samples should also be collected for isolation of circulating tumour cells (CTCs) and circulating plasma DNA (cpDNA) (Fig. 3). Molecular analysis of these samples should be used to stratify patients to treatment with targeted therapies within clinical studies (Fig. 3). In addition, novel aberrations should drive translational studies and drug discovery efforts to identify novel therapeutic strategies for the treatment of SCLC (Fig. 3). This approach is further supported by a recent phase II basket trial that included patients with SCLC. The study failed to demonstrate clinical benefit with targeted therapies based on limited molecular profiling from a single tumour biopsy [23]. We believe this approach will increase our understanding of SCLC, and with the identification and validation of molecular signatures associated with SCLC we may finally see clinical benefit from existing targeted therapies, and see a new wave of therapies developed for the treatment of SCLC.

## Conclusions

This study demonstrates the limitations of performing focused molecular analysis on SCLC biopsies after diagnostic investigations are complete. The paucity of tissue available (57 % of patients), the quality of DNA extracted and the low frequency of aberrations detected (1.7 % of patients) means alternative approaches should be sought. To further understand the biology of SCLC and identify molecular aberrations in patients with SCLC that can be incorporated into treatment algorithms and used to identify novel therapeutic strategies for treatment of SCLC we need to focus on obtaining adequate tissue to carry out these studies. This may be through obtaining more tissue at diagnosis and exploring alternative approaches including CTCs and cpDNA. In addition we should move from focused gene analysis with low frequency molecular changes to wider exome-sequencing and targeted sequencing. These approaches will allow sequential analysis of genomic aberrations that will increase our understanding of the molecular basis of SCLC and help further identify patients that are most likely to respond to targeted therapies.

## Abbreviations

SCLC: small cell lung cancer
FFPE: formalin-fixed paraffin-embedded
ED: extensive stage disease
PCI: prophylactic cranial radiation
VALG: veterans administration lung study group
RT-PCR: real time polymerase chain reaction
CE-SSCA: capillary electrophoresis-single strand conformation analysis
FISH: fluorescent in situ hybridisation
EGFR: epidermal growth factor receptors
ECOG: eastern cooperative oncology group
PS: performance status
NSCLC: non-small cell lung cancer
NGS: next generation sequencing
HTA: human tissue act
CTCs: circulating tumour cells
